# Role of Facial Nerve Reconstruction With Anastomosis and Polyglycolic Acid Tube in Accelerating Functional Recovery After Axotomy in the Rat Facial Nucleus

**DOI:** 10.7759/cureus.57326

**Published:** 2024-03-31

**Authors:** Masao Noda, Ryota Koshu, Yuji Takaso, Chortip Sajjaviriya, Makoto Ito, Takaaki Koshimizu

**Affiliations:** 1 Otolaryngology - Head and Neck Surgery, Jichi Medical University, Shimotsuke, JPN; 2 Pharmacology, Jichi Medical University, Shimotsuke, JPN

**Keywords:** microglial response, neurodegeneration, polyglycolic acid, facial nerve injury, facial nerve

## Abstract

Facial nerve injuries stem from trauma or tumor surgery, triggering neurodegeneration and neuronal cell death in the facial nucleus, consequently inducing irreversible nerve paralysis. Following facial nerve transection, glial cells are activated and undergo proliferation, facilitating motor neuron survival, repair, and regeneration. Clinical approaches, including nerve anastomosis and hypoglossal nerve grafting, require delicate microscopic techniques. Recent advancements involve nerve reconstruction using polyglycolic acid (PGA) tubes, which yield nerve function improvement. However, the central pathophysiological effects of these procedures remain unclear. Therefore, using PGA tubes, we evaluated neurodegeneration and microglial inflammatory response in rats after facial nerve transection. Facial nerve functions were evaluated using vibrissae and blink reflex scores. In the end-to-end anastomosis and PGA tube reconstruction groups, a partial improvement in facial motor function was observed, with increased nerve fiber survival in the former. Approximately 90% of neurons survived in both groups, wherein gliosis exhibited increased microglial activation compared to that in the transection group. These results indicate that PGA tube-assisted nerve reconstruction post-facial nerve transection, although inferior to end-to-end anastomosis, improved certain functions and prevented neuronal cell death. Furthermore, the prolonged inflammatory response in the facial nerve nucleus underscored the correlation between neuronal function and survival and microglia.

## Introduction

Facial nerve transection arises from trauma, such as facial or temporal bone fractures, or from head and neck surgery, such as parotid tumor removal. This condition significantly affects individuals, presenting as facial motor paralysis and consequential facial asymmetry. Following transection, several treatment options are available, including end-to-end anastomosis or anastomosis with other nerves, such as the hypoglossal nerve. These procedures require delicate execution under a microscope to improve function. However, the outcomes of surgical treatments are not consistently satisfactory. Recently, nerve reconstruction techniques using artificial materials such as polyglycolic acid (PGA) tubes have been performed, and this approach has shown some reported functional improvements [1].

In a rat study involving PGA tubes, an interpositional jump graft with end-to-side suture was reported to preserve nerve function; however, the myelinated nerve remained inferior to that of an autograft. Nevertheless, this approach could serve as an effective alternative in cases where nerve material cannot be anastomosed owing to distance or the nature of the nerve [2].

However, the impact of using such artificial materials on the preservation of the central nervous system and changes in glial cells remains unexplored. Additionally, their effectiveness on the central nervous system compared to that of nerve anastomosis is yet to be completely elucidated.

In adult rats, facial nerve injury causes various cellular responses in the facial nucleus. However, owing to the sensitivity of the nerve, its regenerative capacity is limited. Moreover, local glial cells (microglia and astrocytes) are activated as a result of a remote injury to the peripheral nerve axons [3-5]. Metabolic and trophic factors are involved in nerve protection in response to the stimulation of various nerve cleavages [6-8]. These cellular responses are speculated to be mediated by specific signaling pathways following facial nerve transection. Moreover, glial activity and proliferation have been reported to be closely related to motor neuron survival, repair, and regeneration [9,10].

In this study, using facial nerve reconstruction with a PGA tube, we aimed to elucidate nerve preservation and assess glial cell responses in the central nervous system. Additionally, we sought to evaluate the efficacy of this procedure compared to that of nerve anastomosis. This will provide a better understanding of the effectiveness of reconstructive methods actually performed in clinical practice.

## Materials and methods

Animals and surgical procedure

In this study, 24, 9-12-week-old, male Sprague-Dawley rats were used. All animal experiments were performed in accordance with the ARRIVE guidelines and approved by the Animal Care and Use Committee of Kanazawa University (AP-194110).

**Figure 1:**
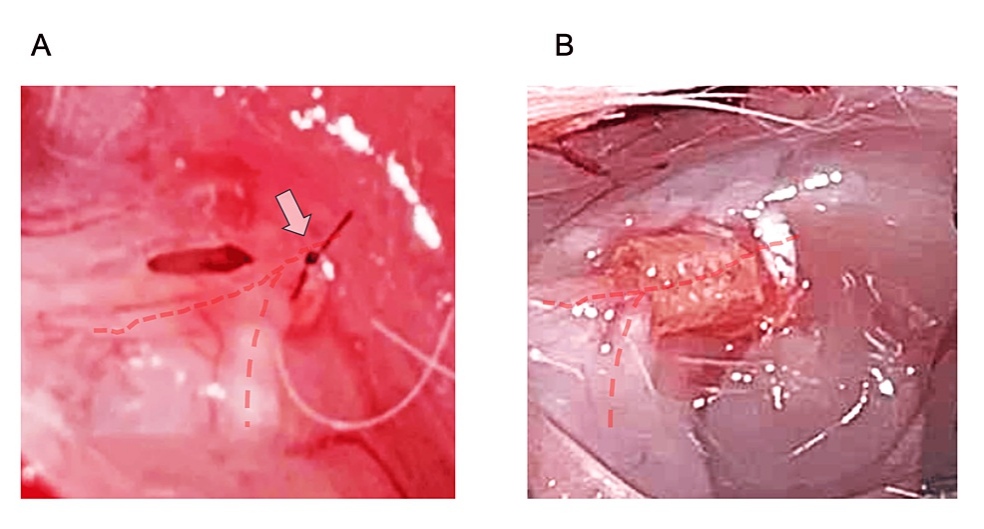
Surgical observations of nerve reconstruction with end-to-end anastomosis and PGA tube reconstruction (A) The left facial nerve trunk was severed as it exited the stylomastoid foramen and the facial nerve was sutured using 10-0 nylon. Red line indicates facial nerve, and white arrow indicates severed and sutured point. (B) The PGA tube was tunneled and each nerve was inserted from both sides. Red line indicates facial nerve. PGA, polyglycolic acid

The surgical procedures were performed as previously described [11]. Systemic anesthesia was induced using a combination anesthetic comprising 0.3 mg/kg of medetomidine (Orion Corporation, Espoo, Finland), 4.0 mg/kg of midazolam (Maruishi Pharmaceutical Co., Ltd., Osaka, Japan), and 5.0 mg/kg of butorphanol (Meiji Seika Pharma Co., Ltd., Tokyo, Japan), injected intraperitoneally. After anesthesia, a postauricular incision was made under a microscope to reveal the peripheral facial nerve at the stylomastoid foramen. For end-to-end anastomosis, the nerves were sutured at two locations using 10-0 nylon (Alfresa, Osaka, Japan) (Figure 1A). For nerve reconstruction using a 1-mm PGA tube, each nerve that was transected at the stylomastoid foreman was inserted from both sides of the collagen-coated PGA tube (Figure 1B).

The animals were euthanized on post-operative day (POD) 7 and 90 through perfusion of 4% paraformaldehyde (PFA); the brainstem and bilateral facial nerve were extracted, post-fixed in 4% PFA, and dehydrated in 30% sucrose. Coronal sections were cut into 17-μm-thick sections using a cryostat (CM1950, Leica, Nussloch, Germany). To evaluate motoneurons in the facial nucleus, the sections were subjected to histopathological staining with cresyl violet (Nissl staining). Images were analyzed using a light microscope (IX83P2-CAS-D8-SP; Olympus, Tokyo, Japan) at 10× magnification, and the number of motor neurons in the facial nucleus was measured using four sections per rat.

Assessment of facial movement

The degree of facial movement was assessed using eye closure and vibrissae movement, according to a previous report (Table 1) [12]. Eye closure was detected through gentle stimulation of the lid margin using a cotton swab, and vibrissae movement was detected via gentle stimulation of the ipsilateral side using a cotton swab; to note, in a normal model where no surgery has been performed, each movement is scored as 3 (totaling 6). This assessment was performed individually on POD7, POD28, and POD90 by two observers.

**Table 1:** Score for rat facial movement Facial movements were evaluated from eye closure capability and motion of vibrissae on the operated left side. Each item was rated on a 4-point scale, with the non-surgical side, the right side, used as the scoring index. Eye closure was detected by gentle stimulation of the lid margin with a cotton swab. Vibrissae movement was detected by gentle stimulation of the ipsilateral side with a cotton swab.

	Eye closure capability
0	No motion
1	Detectable motion
2	Significant (but asymmetric) voluntary motion
3	Symmetric voluntary motion
	Motion of vibrissae
0	No motion
1	Minor trembling
2	Significant (but asymmetric) voluntary motion
3	Effective movement undistinguishable from the contralateral side

Histology

To evaluate axonal degeneration and demyelination, we processed the 14-μm sections for immunostaining using antibodies against myelin basic protein (MBP) (1:400; Merck Millipore, Burlington, MA, USA) and β3-tubulin (1:400; Sigma-Aldrich, St. Louis, MO, USA). To detect astrocytes and microglia in the facial nucleus, we processed the sections for immunostaining using antibodies against glial fibrillary acidic protein (GFAP) (1:400, Sigma-Aldrich, St. Louis, MO, USA) and Iba1 (1:400, Wako Pure Chemical Industries, Osaka, Japan). Alexa 488-conjugated secondary antibodies (1:200, Abcam) or Cy3-conjugated secondary antibodies (1;100, Abcam) were used to visualize the immunolabeling. Imaging was performed using a laser scanning confocal microscope (Eclipse TE200U; Nikon, Tokyo, Japan) with Nikon EZ-C1 or a fluorescence microscope (BZ-X710; Keyence, Osaka, Japan) at 20× magnification. The number of GFAP- and Iba1-positive cells with 4′,6-diamidino-2-phenylindole-positive nuclei in the facial nucleus was measured using four sections per rat. The data are presented as the number of myelinated fibers per area, determined by counting MBP-positive and β3-tubulin-positive nerves. The results for glial cells are presented as the ratio of the number of positive cells in the left facial nuclei to that in the right per area.

Statistical analysis

Statistical analyses were conducted using SPSS Version 26.0 software (IBM Corp., Armonk, NY, USA). Analysis of variance was performed to statistically compare the ratios of neurons, astrocytes, and microglia in the facial nucleus. The p-values for post hoc pairwise comparisons were adjusted using Tukey’s method. Student’s t-test was used to compare the number of myelinated fibers between the end-to-end anastomosis and PGA tube reconstruction model rats. Statistical significance was defined as p < 0.05.

## Results

Facial function recovery following facial nerve reconstruction

**Figure 2:**
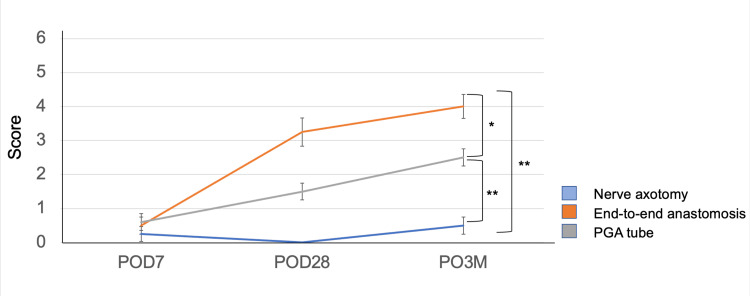
Recovery of facial movement scores in each group The end-to-end anastomosis group exhibited faster and better recovery than the other groups. The PGA tube reconstruction group also exhibited improved outcomes compared to the nerve axotomy group at three months post-surgery. N=4 for each group. PGA, polyglycolic acid; POD, post-operative day

To assess facial function recovery following nerve reconstruction, facial movements were scaled at three different time endpoints (Figure 2).

Over three months, the score for both reconstruction groups showed gradual improvement (F = 28.79, p < 0.0001). In the end-to-end anastomosis and PGA tube reconstruction groups at three months post-operatively, the scores significantly increased from 0.5 (± 0.25) to 4.0 (± 0.35) (p = 0.001) and from 0.6 (± 0.24) to 2.5 (± 0.25) (p = 0.003), respectively. Compared to the PGA tube reconstruction group, the end-to-end anastomosis groups showed significantly better function over the three months (p = 0.037). All individuals showed some level of improvement in function; however, none achieved full recovery. In the axotomy group, little-to-no facial movement was observed.

Neurodegeneration of myelinated fibers and cell death in the facial nucleus following nerve reconstruction with end-to-end anastomosis and PGA tube reconstruction

**Figure 3:**
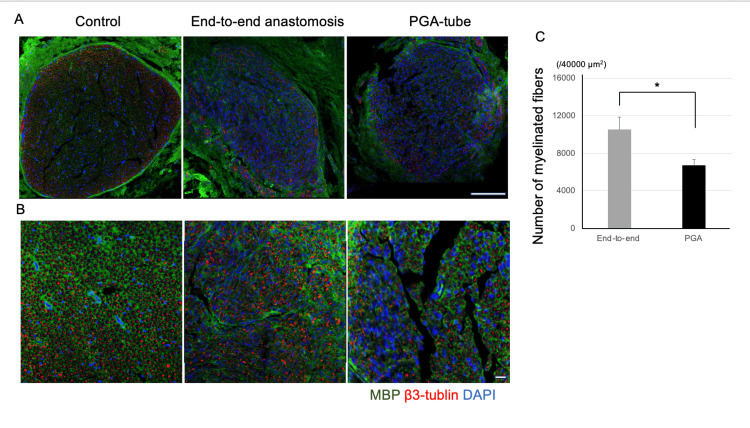
Histological observation and analysis of myelinated fiber using transverse sections of the facial nerve trunk three months post-transaction and suture (A, B) Cross-sections of facial nerves immunostained with MBP (green), β3-tubulin (red), and DAPI (blue). Scale bar: 10 μm (A), magnified view. Scale bar: 100 μm (B). (C) Number of myelinated fibers per area relative to the post-operative date. Data are expressed as mean ± standard error of the mean. N = 4. *p < 0.05; **p < 0.01 as determined based on analysis of variance and post-hoc Tukey’s test. DAPI, 4′,6-diamidino-2-phenylindole; MBP, myelin basic protein

The status of neuronal death in the facial nucleus, as well as the degree of axonal degeneration/demyelination in the facial nerve following reconstruction, was assessed to determine the effect of each nerve reconstruction model. Immunostaining for MBP and β3-tubulin, which stain myelin and axonal structures, allowed the examination of myelinated fibers three months post-nerve reconstruction (Figure 3). Histologically, nerves in the reconstructed group exhibited sparse morphology compared to the non-operated right side (Figure 3A).

In the end-to-end anastomosis group, a significant increase in myelinated axons was observed compared to those in the PGA tube group (p = 0.039).

**Figure 4:**
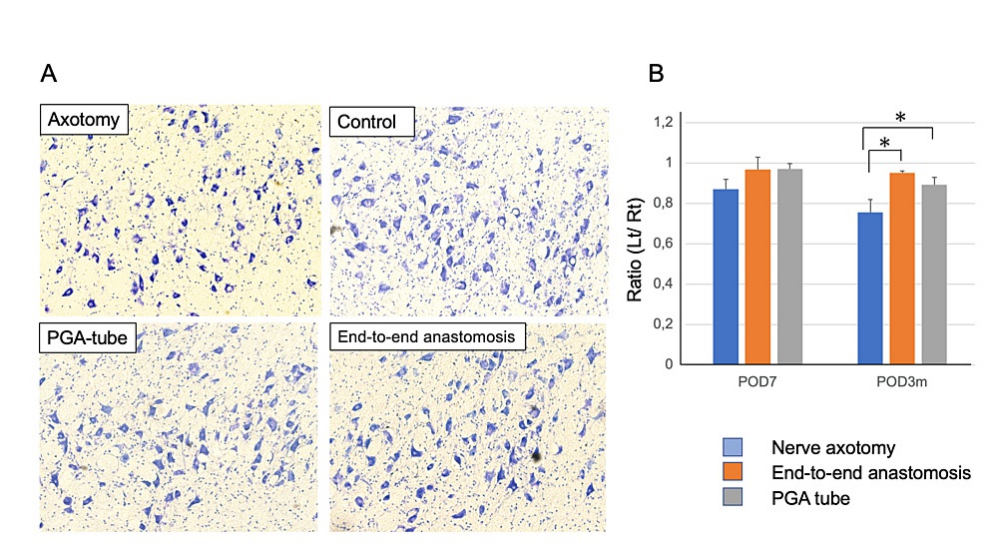
Staining of the facial nucleus with cresyl violet (A) Neurons in facial nuclei were stained with cresyl violet over the course of the experiment. (B) The number of surviving cells in the nucleus is shown at POD7 and at three months post-operatively. POD, post-operative day

Nissl staining revealed that the survival rate of motoneurons in the facial nucleus in each nerve reconstruction group was significantly higher than that in the axotomy group (axotomy group: 75.5 ± 6.45%, end-to-end anastomosis group: 94.9 ± 0.97%, PGA tube group: 89.2 ± 3.5%); however, no significant differences were observed between the end-to-end anastomosis and PGA tube groups up until three months after surgery (Figure 4).

Glial reaction in the facial nucleus following facial nerve reconstruction

**Figure 5:**
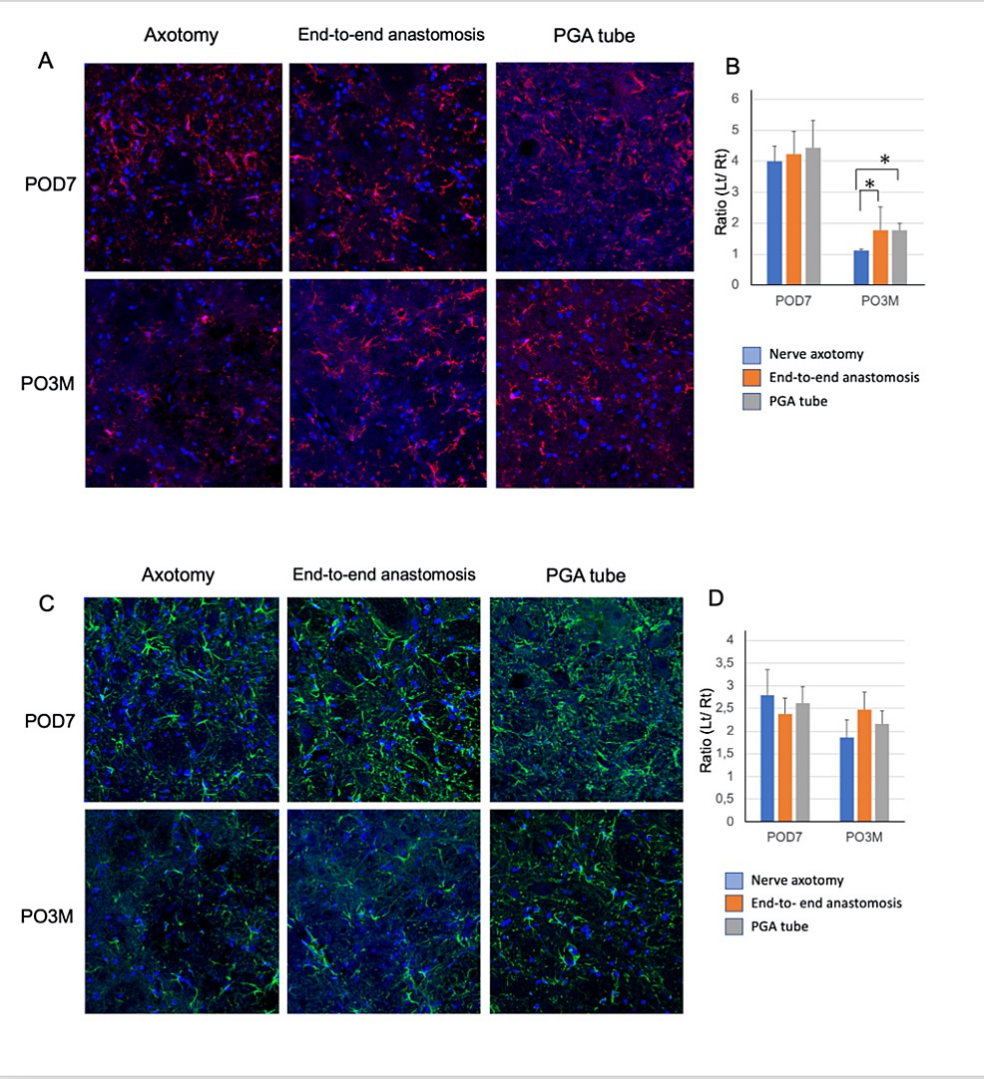
Comparison of time-dependent changes in glial cells between the nerve axotomy and reconstruction groups (A) Facial nuclei from each group immunostained with Iba1 (red) and DAPI (blue) at POD7 and three months post-operatively. (B) Time-dependent changes in the number of Iba1-positive cells. (C) Facial nuclei from each group immunostained with GFAP (green) and DAPI (blue) at POD7 and at three months. (D) Time-dependent changes in the number of GFAP-positive cells. Data are expressed as mean ± SEM. N = 4. *p < 0.05, **p < 0.01, as determined using the t-test. DAPI, 4′,6-diamidino-2-phenylindole; GFAP, glial fibrillary acidic protein; POD, post-operative day

Immunohistochemical analysis revealed that on POD7, the number of Iba1-positive microglia in the facial nucleus was similar across all three groups, achieving a four-fold increase compared to the lateral side. However, a significant increase in these cells was observed in the reconstruction groups up to three months post-surgery (nerve axotomy: 1.127 ± 0.05, end-to-end anastomosis: 1.780 ± 0.22, PGA tube: 1.774 ± 0.21) (Figures 5A, 5B).

Thus, a higher number of Iba1-positive cells in the facial nucleus at three months post-surgery was observed in the nerve anastomosis and PGA tube groups.

Moreover, the number of GFAP-positive astrocytes in the facial nucleus was similar across all groups after axotomy (nerve axotomy: 1.862 ± 0.39, end-to-end anastomosis: 2.475 ± 0.38, PGA tube 2.168 ± 0.28) (Figures 5C, 5D).

## Discussion

In this study, we assessed nerve regeneration and changes in facial nuclei during the reconstruction of facial nerve dissection. The facial nerve in rats was reconstructed using two methods: end-to-end anastomosis and reconstruction with PGA tubes. The results indicated some, albeit incomplete, improvement in facial motor function for both approaches. Additionally, residual nerve fibers and suppression of neuronal cell death were observed over an extended period. PGA tube reconstruction demonstrated some effectiveness; however, it was not as effective as nerve anastomosis. Microglia activation persisted longer in the nerve reconstruction groups than in the group with transection, suggesting a strong association between nerve function and microglia. As a result of anastomosis and immediate reconstruction with PGA following transection, approximately 90% of the nerve cells were preserved. The facial nerve encompasses several components, including motor and autonomic components. However, in this study, the transected nerve primarily consisted of a simple motor component, with cell bodies in the nerve nuclei located in the pons.

Ensuring the prevention of eventual neuronal cell death is a prerequisite for preserving neural function; early anastomosis is deemed important in this regard. However, to reduce the physical shortage of nerves and minimize the surgical invasiveness associated with nerve harvesting, there has been notable progress in the research and development of nerve guide tubes as an alternative to autologous nerve grafts. Of these alternatives, PGA tubes have emerged as a promising option [13,14]. Nerbridge (Toyobo Co., Ltd., Tokyo, Japan) is a PGA tube infused with a collagen sponge, which serves as a scaffold to promote the growth and regeneration of missing nerves. In a previous report on PGA tubes in rats, Niimi et al. utilized collagen-coated PGA tubes with interpositional jump grafts. They demonstrated that the collagen-coated PGA tubes could grow and regenerate sutured facial nerves [2]. Although the autograft group exhibited a greater number of myelinated fibers, the neurological function was comparable [15]. In this instance, a different method of nerve reconstruction was employed. Nevertheless, facial function showed improvement, and the central nerves were also preserved. Previous reports have indicated a correlation between facial function and the number of myelinated nerves [16]; in the present study, the number of myelinated nerve fibers and facial function were lower in the PGA tube group than in the end-to-end anastomosis group. Therefore, despite the effectiveness of PGA, further preservation of the nerve fibers is required.

Furthermore, the nerve reconstruction preserved long-term microglial activation. However, the specific factors that induce and maintain microglia remain unknown. Following facial nerve transection, the CREB/ATF family is altered in neurons, and the phosphorylation of regulatory factors, such as CREB and JNK, is suppressed [17]. During this period, microglia, but not astrocytes, become activated, and the expression levels of CREB, ATF2, and p38 increase, all of which have been shown to play important roles in the central facial nerve. Studies have also indicated that glial cell activation lasts for 10 weeks after reconstruction via anastomosis, and the inhibition of NO production suppresses microglial activity without affecting astrocyte activity [18]. In the present study, microglial activation persisted for an equivalent duration with PGA tubing as it did with anastomosis, suggesting that microglial activation was not dependent on the number of remaining nerve fibers. Although it remains unclear whether microglia are preserved at a certain level of neural activity, it is expected that controlling the nerve-microglia environment will contribute to maintaining high quality of the central nerve system.

In the clinical field, the most common surgical approach for repairing transected nerves involves direct suturing of the two stumps (end-to-end anastomosis); however, functional recovery is often poor [19]. Moreover, in cases where the nerve gap exceeds 5 mm, nerve interposition or “cable graft” procedures are commonly performed. However, the recovery of the transected or resected nerve following reconstruction of the nerve defect remains unsatisfactory [20]. Thus, addressing functional recovery after facial nerve axotomy remains an important significant challenge. Our results suggest a potential avenue for treating facial nerve axotomy, which involves the use of PGA tubes to suppress nerve degeneration. This could prove invaluable, particularly in instances where the surgeon lacks the expertise in nerve suturing or access to microsurgery instruments. Employing PGA tube surgical treatment holds promise as an applicable method to improve the outcomes of facial nerve axotomy.

## Conclusions

This study had a notable limitation. Neuronal cell death occurred in approximately 10% of cases, which, while being significant, also impacts the completeness of facial movements and causes a certain degree of paralysis. This limitation underscores the need for a more effective treatment method, as the current method is not entirely satisfactory.

Our findings demonstrate that PGA can improve facial motor function and reduce neuronal death. It also suggests the potential involvement of microglia in the pathogenesis of the suppression of neuronal death. Therefore, further studies are required to understand the exact role of microglia in the process of nerve degeneration and once the mechanism is defined, microglia-centered countermeasures can be developed to improve the functional recovery after facial nerve axotomy.
